# A Novel Early Memory-Enriched Allogeneic NKG2D CAR-T Cell Therapy Based on CRISPR/Cas9 Technology for Solid Tumors

**DOI:** 10.3390/cancers17193186

**Published:** 2025-09-30

**Authors:** Cristina Aparicio, Mónica Queipo, Marina Belver, Francisco Espeso, Julia Serna-Pérez, Lucía Enríquez-Rodríguez, Carlos Acebal, Álvaro Martín-Muñoz, Antonio Valeri, Alejandra Leivas, Paula Río, Daniel J. Powell, Rosa Lobo-Valentín, David Arrabal, Joaquín Martínez-López, Ana Sánchez, Miguel Á. de la Fuente, Margarita González-Vallinas

**Affiliations:** 1 Unit of Excellence Institute of Biomedicine and Molecular Genetics of Valladolid (IBGM), Higher Council for Scientific Research (CSIC)—University of Valladolid (UVA), 47003 Valladolid, Spain; 2 Department of Biochemistry and Molecular Biology and Physiology, Faculty of Medicine, University of Valladolid, 47003 Valladolid, Spain; 3 Hospital Universitario 12 de Octubre-Centro Nacional de Investigaciones Oncológicas (H12O-CNIO) Haematological Malignancies Clinical Research Unit, Spanish National Cancer Research Centre, 28029 Madrid, Spain; 4 Centro de Investigación Biomédica en Red de Cáncer (CIBERONC), 28029 Madrid, Spain; 5 Department of Hematology, Hospital Universitario 12 de Octubre-Universidad Complutense, Instituto de Investigación Sanitaria Hospital 12 de Octubre (imas12), 28041 Madrid, Spain; 6 Biomedical Innovation Unit, Centro de Investigaciones Energéticas, Medioambientales y Tecnológicas (CIEMAT), 28040 Madrid, Spain; 7 Centro de Investigación Biomédica en Red de Enfermedades Raras (CIBERER), 28029 Madrid, Spain; 8 Instituto de Investigaciones Sanitarias Fundación Jiménez Díaz (IIS-FJD), 28040 Madrid, Spain; 9 Department of Pathology and Laboratory Medicine, University of Pennsylvania, Philadelphia, PA 19104-4238, USA; 10 Clinical Genetics Laboratory, Río Hortega University Hospital, 47012 Valladolid, Spain; 11 Citospin S.L., Parque Científico Universidad de Valladolid, 47011 Valladolid, Spain; 12 Department of Cell Biology, Genetics, Histology and Pharmacology, Faculty of Medicine, University of Valladolid, 47003 Valladolid, Spain

**Keywords:** chimeric antigen receptor (CAR), T cells, allogeneic, off the shelf, solid tumors, T-cell memory, CRISPR, NKG2D, interleukins, cancer immunotherapy

## Abstract

**Background/Objectives:** Chimeric Antigen Receptor (CAR)-T cell therapy has demonstrated impressive clinical results against hematological malignancies. However, currently commercialized CAR-T therapies are designed for autologous use, which entails some disadvantages, including high costs, manufacturing delays, complex standardization, and frequent production failures due to patient T cell dysfunction. Moreover, their CARs target one specific antigen, increasing the probability of antigen-negative tumor relapses. To overcome these limitations, we developed a novel NKG2D CAR-T cell therapy for allogeneic use with broad target specificity, as this CAR targets eight different ligands commonly upregulated in both solid and hematological tumors. Additionally, the manufacturing process was optimized to improve the phenotypic characteristics of the final product. **Methods:** Multiplex CRISPR/Cas9 technology was applied to eliminate the expression of TCR and HLA class I complexes in healthy donor T cells to reduce the risk of graft-versus-host disease and immune rejection, respectively, as well as lentiviral transduction for introducing the second-generation NKG2D-CAR. Moreover, we sought to optimize this manufacturing process by comparing the effect of different culture interleukin supplementations (IL-2, IL-7/IL-15 or IL-7/IL-15/IL-21) on the phenotypic and functional characteristics of the product obtained. **Results**: Our results showed that the novel CAR-T cells effectively targeted cervicouterine and colorectal cancer cells, and that those manufactured with IL-7/IL-15/IL-21 supplementation showed the most suitable characteristics among the conditions tested, considering genetic modification efficiency, cell proliferation, antitumor activity and proportion of the stem cell memory T cell subset, which is associated with enhanced in vivo CAR-T cell survival, expansion and long-term persistence. **Conclusions:** In summary, this new prototype of NKG2D CAR-T cell therapy for allogeneic use represents a promising universal treatment for a wide range of tumor types.

## Simple Summary

Chimeric Antigen Receptor (CAR)-T cell therapy has shown significant success in treating hematological cancers, but commercialized autologous CAR-T therapies face challenges such as high costs, manufacturing delays, complex standardization and the risk of tumor relapses due to single-antigen targeting. To address these issues, a novel allogeneic CAR-T therapy with broader target specificity was developed, optimizing its manufacturing process. Using CRISPR/Cas9, TCR and HLA class I complex expression were eliminated from donor T cells to reduce the risk of immune rejection and graft-versus-host disease. Additionally, NKG2D CAR, targeting eight ligands upregulated in both solid and hematological tumors, was lentivirally transduced. This study optimized CAR-T cell manufacture by testing various interleukin supplements (IL-2, IL-7/IL-15, IL-7/IL-15/IL-21). Results showed that IL-7/IL-15/IL-21 supplementation produced CAR-T cells with the most suitable characteristics in terms of genetic modification efficiency, cell proliferation, antitumor activity and memory profile. This new allogeneic NKG2D CAR-T therapy represents a promising universal treatment for a variety of cancers.

## 1. Introduction

Chimeric Antigen Receptor (CAR)-T cell therapy has emerged as one of the most promising therapeutic strategies due to its impressive clinical results even in patients with relapsed or refractory advanced tumors [1]. So far, all CAR-T cell therapies approved for commercialization are designed to target only hematological malignancies and manufactured from the patients’ own T cells (i.e., autologous) [2]. Consequently, although they have been used to successfully treat thousands of patients, current CAR-T cell therapies have several limitations such as low quality and quantity of the starting material due to the disease and/or previous treatments, high costs that limits their worldwide accessibility, manufacturing delays which are crucial for patients with progressive disease, complex standardization of the final product due to donor variability, high probability of production failure because the final product is not achieved or does not comply with specifications, and risk of product contamination with tumor cells. Therefore, some studies focus on anticipating and ameliorating these issues to improve product manufacture and patient outcome [3,4,5,6].

The use of allogeneic CAR-T cells, manufactured from donor T cells, represents a promising alternative that could address these issues. However, this type of therapy also has two major challenges: avoiding the risk of graft-versus-host disease (GvHD) mediated by T-cell receptor (TCR) αβ signaling of the CAR-T cells and the graft rejection due to the recognition of the human leukocyte antigen (HLA)-mismatched donors by the host immune cells, thus limiting its persistence [7,8]. In order to overcome these drawbacks, several allogeneic CAR-T cell strategies have been recently reported, such as genetically modified T cells, virus-specific T cells, γδ T cells, umbilical cord blood-derived T cells, and induced pluripotent stem cells (iPSC)-derived T cells among others [9,10]. Among them, the most advanced strategy in clinical research is the one based on genetically modified CAR-T cells, since some products of this type are in phase 2 clinical trials (e.g., WU-CART-007 [NCT06514794]). However, virus-specific CAR-T cells, γδ CAR-T cells and iPSC-derived CAR-T cells are currently in phase 1 clinical trials (e.g., CTM-N2D [NCT05302037], CD30.CAR-EBVST [NCT04288726] and FT819 [NCT04629729], respectively), and the umbilical cord blood-derived CAR-T cells have not yet reached clinical development.

One strategy to avoid GvHD is the genetic editing of T cells to eliminate the expression of the TCR complex. This may be performed by using clustered regularly interspaced short palindromic repeats/Cas9 protein (CRISPR/Cas9) technology. In comparison with other gene-editing technologies, such as transcription activator-like effector nucleases (TALEN) or zinc finger nucleases (ZFN), CRISPR/Cas9 technology is usually preferred due to its capacity for multiple simultaneous gene editing, high efficiency and flexibility, and also because it uses less labor, and is cheaper and more scalable [11,12]. Indeed, this technology was the basis for the first FDA-approved gene editing therapy, which was exagamglogene autotemcel (Casgevy^TM^), a treatment for sickle cell disease and transfusion-dependent β-thalassemia [13].

One of the most important hurdles associated with CAR-T cell treatments is the process of tumor antigen escape, usually caused by partial or complete loss of the targeted antigen, leading to treatment resistance and subsequent disease relapse and recurrence [14,15]. This is relatively frequent because CARs are typically able to recognize a single antigen, and in conjunction with the antigenic heterogeneity present within the same tumor, especially in solid tumors, further increases the probability of antigen escape [14,16,17]. In addition, the recognition of a single tumor antigen by the CAR usually restricts its use to a limited subset of tumor types. Furthermore, due to the lack of tumor-specific antigens in solid tumors, CAR-T cells are often designed to target tumor-associated antigens that are also present in normal cells, which can cause on-target off-tumor effects [18]. Therefore, the search for a CAR that is able to recognize different tumor antigens and whose targets are expressed on different tumor types, but generally absent in normal cells, is crucial to achieve a more universal cell therapy.

One potentially promising CAR with broad target specificity is based on the natural-killer group 2 member D (NKG2D) receptor, which is a type II transmembrane-anchored C-type lectin-like protein activating receptor, typically expressed on natural killer (NK) cells and some specific T-cell subsets (NK T cells, subpopulations of γδ T cells, CD8+ T cells, and some autoreactive CD4+ T cells) [19,20]. This receptor is able to recognize eight different ligands (NKG2D-L) represented by two MHC class I-related proteins (MICA and MICB) and six UL16-binding proteins (ULBP1-6) [19,20,21]. Those ligands are generally absent in healthy tissues under homeostatic conditions, thus preventing self-recognition and autoimmune reactivity, but are upregulated in both solid and hematological tumors [21,22]. This type of CAR has been described as safe and effective in mouse models [20,23], and is currently used in some ongoing clinical trials [24].

A strategy to improve the clinical antitumor efficacy of the CAR-T cells is to promote the early memory T-cell phenotype, which includes stem cell memory (Tscm) and central memory (Tcm) T cell subsets. A higher proportion of these less differentiated T cells in the CAR-T-cell product has been correlated with improved survival, expansion and long-term persistence of the therapeutic cells after infusion due to their higher proliferation capacity, self-renewal, stemness and lower exhaustion profile [25,26]. In contrast, in CAR-T-cell products with a high proportion of more differentiated T cells, such as the effector memory (Tem) and effector (Teff) T-cell subsets, their cytotoxic capacity is increased while their proliferation, memory function and lifespan are decreased [25,26]. During ex vivo T-cell expansion, maintenance of early memory T-cell phenotype can be promoted by specific interleukin (IL) supplementation in the culture media [27]. Traditionally, IL-2 has been the standard IL used in the manufacturing process, as it greatly enhances CAR-T-cell proliferation, but at high concentrations, it also produces exhaustion and a high differentiation profile, decreasing memory T-cell formation [27,28]. Therefore, other ILs such as IL-7, IL-15 and/or IL-21 have recently been studied as promising alternatives to promote the enrichment of early memory T cells [27,29,30].

In the present study, we developed a manufacturing process to produce a novel allogeneic NKG2D-CAR-T-cell therapy by eliminating TCR and HLA class I complexes using CRISPR/Cas9 technology, thus avoiding the risk of GvHD and allorejection, respectively. The use of the atypical NKG2D-CAR, based on the ligand-binding domain of the NKG2D receptor, which targets eight different ligands upregulated in more than 70% of all types of cancer, including both solid and hematological tumors [22], confers a wide therapeutic range to the novel CAR-T cells with less probability of therapy resistance. Furthermore, we optimized the procedure by analyzing the use of different IL supplementation conditions (IL-2, IL-7/-15 and IL-7/-15/-21), in order to determine the best option to enrich the Tscm cell subset in our product, while maintaining a high antitumor effect.

## 2. Materials and Methods

### 2.1. Buffy Coat Samples

Buffy coat samples were provided by the regional blood bank, Centro de Hemoterapia y Hemodonación de Castilla y León (CHEMCYL; Valladolid, Spain), with the previous informed consent of the healthy donors and the approval of the corresponding Ethics Committee.

### 2.2. Peripheral Blood Mononuclear Cell (PBMC) Isolation and T-Cell Purification

Human PBMCs were isolated from buffy coats of healthy donors by using Ficoll-Paque^TM^ Plus (17144002, Cytiva, Uppsala, Sweden) density gradient. Firstly, 20 mL Ficoll was added, followed by 30 mL of diluted buffy coat (dilution with PBS containing 2.5% human serum albumin, HSA, 1:1 ratio). Then, it was centrifuged at 400× *g* for 30 min without a brake and the band of PBMCs was isolated. The PBMCs were washed twice with PBS containing 2.5% HSA and centrifuged at 300× *g* for 10 min. CD3-positive cells (T cells) were purified from the PBMCs using EasySep™ Release Human CD3-Positive Selection Kit (17751, StemCell Technologies, Vancouver, BC, Canada) according to the manufacturer’s protocol and were stimulated with 25 µL/mL ImmunoCult™ Human CD3/CD28 T Cell Activator (10971, StemCell Technologies). The activated T cells were cultured in RPMI 1640 Medium + GlutaMAX^TM^-l (61870-010, Gibco, Waltham, MA, USA) with 10% fetal bovine serum (FBS) (SV30160.03, Cytiva) in the presence of different types of interleukins: 50 IU/mL IL-2 (130-097-746, Miltenyi Biotech, Bergisch Gladbach, Germany), 50 IU/mL IL-7 (200-07, Peprotech, Cranbury, NJ, USA), 50 IU/mL IL-15 (200-15, Peprotech), and/or 50 IU/mL IL-21 (200-21, Peprotech) and maintained in a humidified atmosphere (>95%) at 37 °C and 5% CO_2_.

### 2.3. Cell Lines and Culture

Established tumor cell lines used were HeLa (CCL-2, ATCC), HCT-116 (CCL-247, ATCC) and HT29 (HTB-38, ATCC) that were lentivirally transduced with the vector pSin-GFP (Addgene, Watertown, MA, USA) and selected with puromycin (1 µg/mL HeLa and HT29 and 0.8 µg/mL HCT-116) for 5 days, thus obtaining 96% of GFP-expressing cells. In addition, 293FT cells (R70007, ThermoFisher Scientific, Waltham, MA, USA) were used for lentiviral production.

These cell lines were cultured in DMEM + GlutaMAX^TM^-l (31966-021, Gibco) supplemented with 10% FBS and antibiotics (100 U/mL penicillin and 100 µg/mL streptomycin, 15140-122, Gibco), and maintained in a humidified atmosphere (>95%) at 37 °C and 5% CO_2_.

### 2.4. Flow Cytometry

Cells (1 × 10^5^) were centrifuged for 5 min at 400× *g*, washed with 1 mL PBS and centrifuged again. Washed cells were then resuspended with 1 mL PBS, and we added the viability reagent (LIVE/DEAD Fixable Violet Dead Cell Stain Kit) and incubated for 5 min at room temperature in the dark. Then, the cells were centrifuged and resuspended in 100 µL PBS with the appropriate dilution of the corresponding antibodies and stained for 15 min at room temperature in the dark. Immediately after washing, data were acquired on the Gallios (Beckman Coulter, Brea, CA, USA) or Cytek Aurora (Cytekbio, Fremont, CA, USA) and analyzed by Kaluza software 2.2 (Beckman Coulter) or FlowJo version 7.0 (Tree Star, Inc., Ashland, OR, USA).

The following antibodies and reagents were used in flow cytometry analysis: from Immunostep (Salamanca, Spain), anti-CD314-APC (clone 1D11, 314A-100T), anti-CD62L-PE (clone HI62L, 62LPE-100T), anti-CD3-FITC (clone 33-2A3, 3F1-100T), anti-CD4-PerCP Cyanine 5.5 (clone HP2/6, 4PPC5.5-100T); from Invitrogen (Waltham, MA, USA), anti-CD45RO-APC (clone UCHL1, 17-0457-41), LIVE/DEAD Fixable Violet Dead Cell Stain Kit (L34963); from BD Biosciences (Franklin Lakes, NJ, USA), anti-MICA-BUV805 (Clone 159227, 749768), anti-CD8-APC Cyanine 7 (Clone SK1, 557834), anti-CD95-PE-CF594 (Clone DX2, 562395), anti-CD45RA-PE Cyanine 7 (HI100, 560675); from R&D Systems (Minneapolis, MN, USA), anti-ULBP 2/5/6-Alexa Fluor 700 (Clone 165903, FAB1298N-100UG), anti-ULBP1-Alexa Fluor 405 (Clone 170818, FAB1380V), anti-ULBP3-PE (Clone 165903, FAB1517P), anti-MICB-APC (Clone 236511, FAB1599A-100); from Santa Cruz Biotechnology (Dallas, TX, USA), anti-ULBP4-Alexa Fluor 594 (clone 6E6, sc-53133 AF594); from eBioscience^TM^ (San Diego, CA, USA), anti-HLA-ABC-FITC (clone W6/32, 11-9983-42); from ThermoFisher Scientific, anti-TCR Alpha/beta-PE (clone IP26, MA1-10455). For the analyses, Fluorescence Minus One (FMO) controls were performed to define the boundaries of the populations and to establish the appropriate panel compensation.

### 2.5. CRISPR/Cas9-Mediated Double-Gene Knockout in T Cells and TCR-Negative Enrichment

Synthesis and purification of guide RNAs (gRNAs) was performed with GeneArt™ Precision gRNA Synthesis Kit (A29377, Invitrogen) according to the manufacturer’s instructions. gRNAs targeting constant regions of TCRα (*TRAC*) and β-2 microglobulin (*B2M*) were used to eliminate the expression of TCR and HLA class I (HLA-I) complexes, respectively (Table 1). gRNA selection was performed to minimize off-target effects by considering the scores obtained from in silico analyses (target scores: −76.8 [TRAC] and −80 [B2M] [31]).

A total of 1 × 10^6^ activated T cells were electroporated using Neon™ Transfection System 100 µL Kit (MPK10096, ThermoFisher Scientific) at 3 pulses of 1600 V and 10 ms. To generate *TRAC* and *B2M* knockout T cells, gRNAs targeting the respective genes (0.77 µM each) were co-electroporated with 5 µg TrueCut™ Cas9 Protein v2 (A36499, Invitrogen). Electroporated cells were immediately placed in the specific culture medium and cultured at 37 °C and 5% CO_2_. The electroporation efficiency was determined by flow cytometry analysis.

In order to enrich the TCR-negative T cells, the T cells were negatively selected using the EasySep™ Release Human CD3-Positive Selection Kit by modifying the protocol to collect the flow-through fraction.

### 2.6. Lentiviral Production and Transduction

The CAR construct used in this study is a second-generation CAR that consists of NKG2D binding domain, CD8α hinge and transmembrane domain, 4-1BB co-stimulatory domain and CD3ζ intracellular domain (see details in [32]).

Lentiviral vectors were produced in 293FT cells, cultured with DMEM + GlutaMAX^TM^-l supplemented with 10% FBS, with a solution mix containing the second-generation lentiviral packaging plasmids (7.5 µg psPAX2 [12260, Addgene], 5 µg pMD2.G [12259, Addgene]) and 10 µg target vector, 67.5 µg polyethylenimine (043896.01, ThermoFisher) and 1 mL OPTI-MEM (11520386, Gibco). After vortexing the mixture for 5 s and incubating for 20 min at room temperature, it was added to the 293FT cells seeded in a 75 cm^2^ flask (12 mL of medium, around 80–90% confluency) for transfection. The next day, the medium was changed and the following day the viral supernatant was filtered, frozen at −80 °C, and new medium was added to the cells. The next day, the viral supernatant was also filtered and lentiviral particles from the collected medium were concentrated by ultracentrifugation for 2 h at 64,000× *g*. The pellet was dried for 1 h and then 1 mL of PBS containing 1% HSA was added and incubated overnight at 4 °C. Finally, the viruses were aliquoted and frozen at −80 °C until use.

Lentiviral vectors were validated by transducing 5 × 10^5^ T cells with serial dilutions and quantifying NKG2D-CAR expression by flow cytometry, obtaining a viral titer of 4.8 × 10^7^ transducing units (TU) per mL of our stock. The lentiviral transduction was performed by adding the lentiviral vector to T cells with an MOI of 9.7, and they were cultured in RPMI 1640 Medium + GlutaMAX^TM^-l supplemented with 10% FBS and in the presence of different interleukin supplementation.

### 2.7. In Vitro Cytotoxicity Assay

Antitumor efficacy of CAR-T cells was evaluated by co-culturing them with GFP-positive tumor cell lines derived from cervicouterine (HeLa) and colorectal (HCT116 and HT29) cancers. Tumor cells (12,500 cells of HT29 and 10,000 cells of HeLa and HCT116) were seeded in 96-well flat-bottom tissue-culture plates with DMEM + GlutaMAX^TM^-l supplemented with 10% FBS. After 24 h, the medium was removed and T cells were added with RPMI 1640 Medium + GlutaMAX^TM^-l supplemented with 10% FBS to the plates at an effector-to-target (E:T) ratio of 4:1. After 72 h, the medium was removed and replaced by PBS to avoid the background of the dead cells, and the quantification of fluorescence, the readout of tumor cell viability, was performed with Cytation 5 (BioTek, Santa Clara, CA, USA). The percentage of tumor cell viability was calculated as follows: coculture fluorescence × 100/tumor cells fluorescence (control).

### 2.8. ELISA Assay

Cytokine release by CAR-T cells was evaluated by co-culturing with tumor target cell lines at a 4:1 ratio for 72 h with RPMI 1640 Medium + GlutaMAX^TM^-l supplemented with 10% FBS. Supernatants were collected and analyzed by ELISA to measure the IFN-γ, TNFα and IL-2 levels (DY285, DY210 and DY202, respectively, R&D Systems) following the manufacturer’s protocol.

### 2.9. Karyotype Analysis

Karyotyping was performed following the ISO 15189:2022 standard in an accredited laboratory. To that end, 100 µL Colcemid solution (9311, FUJIFILM Irvine scientific, Santa Ana, CA, USA) and 100 µL 10% Ethidium Bromide solution 1% (A1152,0025, PanReac AppliChem ITW Reagents, Barcelona, Spain) were added to 2 × 10^6^ T cells in 5 mL of RPMI 1640 Medium + GlutaMAX^TM^-l supplemented with 10% FBS. After an incubation for 20 min at 37 °C, it was centrifuged for 8 min at 688× *g*, and the pellet was resuspended with 6 mL of hypotonic (0.075 M) KCl solution (L0643-500, Biowest, Nuaillé, France) and incubated for 22 min at 37 °C. To fix the cells, 1 mL Carnoy’s solution (1:3 acetic acid:methanol solution) was added, and the cells were washed a total of 4 times with 6 mL Carnoy’s solution by centrifugating for 8 min at 688× *g*. After the final centrifugation, the cells were resuspended with 500 µL Carnoy’s solution, and 3–4 drops of culture were dispensed onto a slide and dried vertically at room temperature. For G-banding, the slides were heated for 18 h at 60–67 °C and then placed in 0.25% trypsin solution (215240, BD). After 12 s, the slide was washed with distilled water and stained with Leishman solution (1 mL Leishman stock solution, consisting of 150 mg Leishman’s eosin methylene blue (1.01350.0010, Merck, Darmstadt, Germany) dissolved in 100 mL methanol, and 3 mL Gurr buffer) for 3 min. Next, it was washed with Gurr buffer at pH 6.8 (1.11374.0100, Merck) and dried at room temperature. The metaphase spreads were captured with an Axio Imager Z2 (Zeiss, Oberkochen, Germany) and analyzed with the Ikaros software (version 5.3.6).

### 2.10. Statistics

Statistical analyses were performed using IBM SPSS Statistics 26 version, and the results were presented as mean ± SEM of at least 4 independent experiments, unless otherwise indicated. Data sets were analyzed with Shapiro–Wilk for normality and Levene test for homogeneity of variances. Parametric data were analyzed with *t*-Student test (2 groups) or one-way ANOVA using Tukey’s post hoc test (≥3 groups). Non-parametric data were analyzed with Mann–Whitney U test (2 groups) or Kruskal–Wallis H test (≥3 groups). A *p* value inferior to 0.05 was considered statistically significant, which was represented as follows: * *p* < 0.05; ** *p* < 0.01; *** *p* < 0.001; **** *p* < 0.0001.

## 3. Results

### 3.1. Simultaneous Disruption of TCR and HLA Class I Expression Using CRISPR/Cas9 Technology

**Figure 1:**
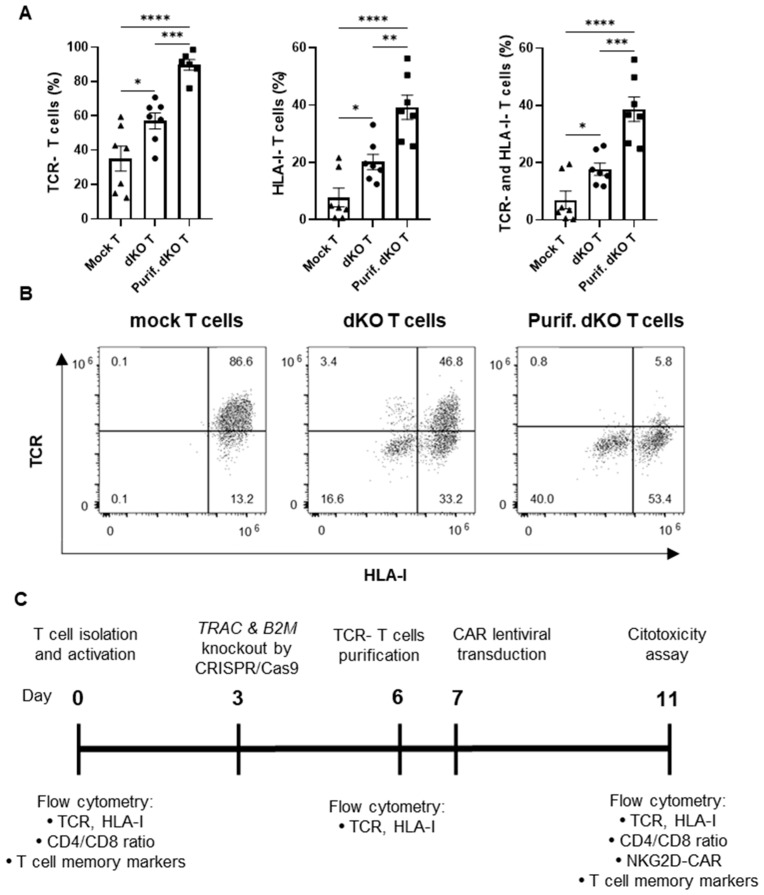
Knockout of TCR and HLA class I complexes in T cells by CRISPR/Cas9 technology: (**A**) Percentage of TCR-negative, HLA-I-negative or both TCR-negative and HLA-I-negative T cells in mock T cells (Mock T), double-knockout (*TRAC* and *B2M* genes) T cells before CD3-negative enrichment (dKO T) and double-knockout T cells after CD3-negative enrichment (purified dKO T cells, Purif. dKO T) in T cell cultures supplemented with IL-2, 3 days after genetic modification (day 6; *n* = 7 independent experiments with different donors). (**B**) Representative dot plots of the TCR and HLA class I knockout analysis by flow cytometry. (**C**) Timeline representation of the experimental design for the production and testing of allogeneic NKG2D CAR-T cells. Data are shown as mean ± SEM. Statistical significance is represented as: * *p* < 0.05, ** *p* < 0.01, *** *p* < 0.001, **** *p* < 0.0001.

In adoptive cell therapy with CAR-T cells for allogeneic use, it is imperative to minimize the risks of GvHD and allorejection. To this end, we applied CRISPR/Cas9 ribonucleoprotein (RNP) technology to simultaneously knock out the *TRAC* and *B2M* genes, aiming to eliminate the expression of TCR and HLA-I complexes. The CRISPR/Cas9 RNP complexes were electroporated into the isolated T cells cultured with IL-2 supplementation, 3 days after activation by CD3/CD28 stimulation. The expression of TCR and HLA-I was measured 3 days after the electroporation (day 6 after T cell activation) by flow cytometry, showing more than 55% of TCR-negative cells and 20% of HLA-I-negative cells, and around 20% of double-negative (TCR−/HLA-I−) T cells (Figure 1A,B).

To increase the proportion of TCR-negative T cells in our product, we eliminated CD3+ cells by selection with magnetic beads, 3 days after T-cell electroporation (day 6; Figure 1C), thus enriching the TCR-negative cell population to more than 89%. Likewise, the percentage of HLA-I-negative cells improved significantly, reaching more than 39%. Finally, double-negative T cells accounted for more than 38% of the modified T-cell population (Figure 1A,B).

### 3.2. Generation and Characterization of Allogeneic CAR-T Cells Against NKG2D Ligands

**Figure 2:**
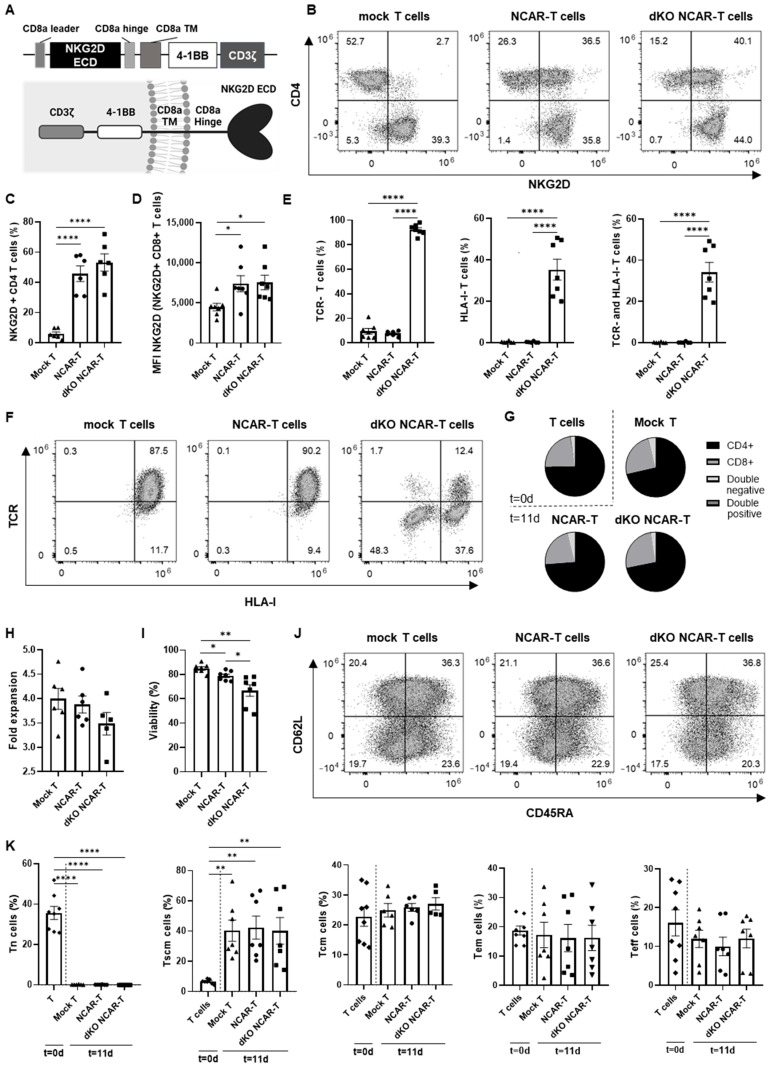
Generation and characterization of allogeneic NKG2D CAR-T cells: (**A**) Representation of the lentiviral NKG2D-CAR construct (up) and the NKG2D-CAR conformation in the membrane (down; created with Biorender.com). NKG2D ECD: NKG2D extracellular domain (amino acids 82–216); TM: transmembrane; CD8a: CD8 alpha. (**B**) Representative dot plots of the NKG2D-CAR expression analysis by flow cytometry. (**C**) Percentage of NKG2D-CAR expressing cells in mock T cells (Mock T), NKG2D CAR-T cells (NCAR-T) and double-knockout (*TRAC* and *B2M* genes) NKG2D CAR-T cells (dKO NCAR-T) manufactured with IL-2 supplementation. Since CD8-positive cells express endogenous NKG2D receptor, NKG2D-CAR expression was determined in the CD4-positive subpopulation. (**D**) Median Fluorescence Intensity (MFI) of NKG2D in flow cytometry analysis of NKG2D- and CD8-positive mock T cells, NKG2D CAR-T cells and double-knockout NKG2D CAR-T cells. (**E**) Percentage of TCR-negative, HLA-I-negative or both TCR-negative and HLA-I-negative T cells in mock T cells, NKG2D CAR-T cells and double-knockout NKG2D CAR-T cells. (**F**) Representative dot plots of the TCR and HLA-I knockout analysis. (**G**) Distribution of T-cell populations according to CD4 and CD8 expression, in generated CAR-T cells and starting T cells (day 0). (**H**) Comparison of fold expansion of mock T cells, NKG2D CAR-T cells and double-knockout NKG2D CAR-T cells after 4 days of culture from NKG2D-CAR transduction (from day 7 to 11). (**I**) Viability of mock T cells, NKG2D CAR-T cells and double-knockout NKG2D CAR-T cells. (**J**) Representative example of analysis of T-cell memory subpopulations by flow cytometry (**K**) Comparison of the different T-cell memory subpopulations of double-knockout NKG2D CAR-T cells with controls and starting T cells (day 0). Unless otherwise indicated, all analyses were performed 11 days after manufacturing initiation. TCR−: TCR negative. HLA-I−: HLA-I negative. Tn: Naïve T cells (CD62L+ CD45RA+ CD95−). Tscm: Stem cell memory T cells (CD62L+ CD45RA+ CD95+). Tcm: Central memory T cells (CD62L+ CD45RA− CD95+). Tem: Effector memory T cells (CD62L− CD45RA− CD95+). Teff: Effector T cells (CD62L− CD45RA+ CD95−). Data are shown as mean ± SEM (*n* = 7 independent experiments with different donors). Statistical significance is represented as: * *p* < 0.05, ** *p* < 0.01, **** *p* < 0.0001.

To obtain the CAR-T cells for allogeneic use, we combined the knockout of the *TRAC* and *B2M* genes with the lentiviral transduction of the CAR. We used an NKG2D-CAR construct, based on the ligand-binding domain of the NKG2D receptor and a second-generation CAR backbone containing 4-1BB and CD3ζ intracellular signaling domains (Figure 2A). One day after CD3-negative T-cell enrichment (day 7), T cells were transduced with an MOI of 9.7, and the efficiency (NKG2D-CAR expression) was measured by flow cytometry 4 days later (day 11). The proportion of NKG2D-CAR-positive T cells was determined by a quantification of NKG2D expression through flow cytometry in the CD4+ T-cell subset, as the CD8+ T cells express the NKG2D receptor endogenously (Figure 2B). The results showed that CAR-positive cells accounted for 53.2 ± 5.7% and 45.7 ± 5.2% of double-knockout NKG2D CAR-T and NKG2D CAR-T-cell populations, respectively (Figure 2C). Moreover, transduction efficiency was also confirmed by the increase in the median fluorescence intensity (MFI) of NKG2D observed in CD8+ T cells (Figure 2D).

Regarding the percentage of TCR-negative, HLA-I-negative or double-negative cells at day 11 after activation, we observed that 92.3 ± 1.6% of the modified cells were TCR-negative, 35.3 ± 5.1% were HLA-I-negative and 34.2 ± 4.8% were double-negative (Figure 2E,F).

Moreover, we studied the CD4/CD8 ratio by analyzing CD4 and CD8 expression by flow cytometry. We found no significant changes in the CD4+/CD8+ distribution between the starting T-cell population (day 0) and the double-knockout NKG2D CAR-T cells after culturing with IL-2 for 11 days, showing a CD4/CD8 ratio around 3:1. Moreover, we confirmed that neither the lentiviral transduction of the CAR nor the electroporation to eliminate TCR and HLA-I expressions by CRISPR/Cas9 significantly affected the CD4/CD8 ratio (one-way ANOVA; *p* > 0.05) (Figure 2G).

In addition, we measured the proliferation capacity of the obtained double-knockout NKG2D CAR-T cells by determining fold expansion during 4 days of culturing (from NKG2D-CAR transduction on day 7 to the end of the manufacturing process on day 11) and observed that there were no significant differences between these cells and the NKG2D CAR-T cells (expressing both TCR and HLA-I) or the mock T cells (Student’s *t*-test; *p* = 0.197 and *p* = 0.081, respectively) (Figure 2H). In terms of viability, a slight decrease is observed in NKG2D CAR-T cells compared to mock T cells, while the decrease in double-knockout NKG2D CAR-T cells is more pronounced (Figure 2I).

As we mentioned above, the in vivo persistence and efficacy of CAR-T-cell therapy is enhanced by a high proportion of early memory T cells, especially Tscm cells. Therefore, we analyzed the different memory subpopulations of the double-knockout NKG2D CAR-T cells by flow cytometry, as shown in Figure 2J (the general gating strategy is illustrated in Figure S1). Results, summarized in Figure 2K, indicated that the proportion of naïve T cells was significantly decreased at the end of the manufacturing process (day 11), which included IL-2 supplementation, while the proportion of Tscm cells was significantly increased. Regarding the proportion of Tcm cells, Tem cells and Teff cells, there were no significant differences (one-way ANOVA; *p =* 0.347; *p =* 0.838; *p =* 0.357, respectively). Since changes were similar in all conditions (mock T cells, CAR-T cells, and double-knockout CAR-T cells), without detecting significant differences between them, we concluded that the proportion of T cell memory subsets is not affected by the genetic modifications of the manufacturing process. The double-knockout NKG2D CAR-T-cell product obtained was enriched in early memory phenotype T cells (40.2 ± 8.8% of Tscm and 27.0 ± 2.1% of Tcm cells).

### 3.3. In Vitro Antitumor Activity of the Allogeneic NKG2D CAR-T Cells Against Human Cervical and Colorectal Tumor Cell Lines

**Figure 3:**
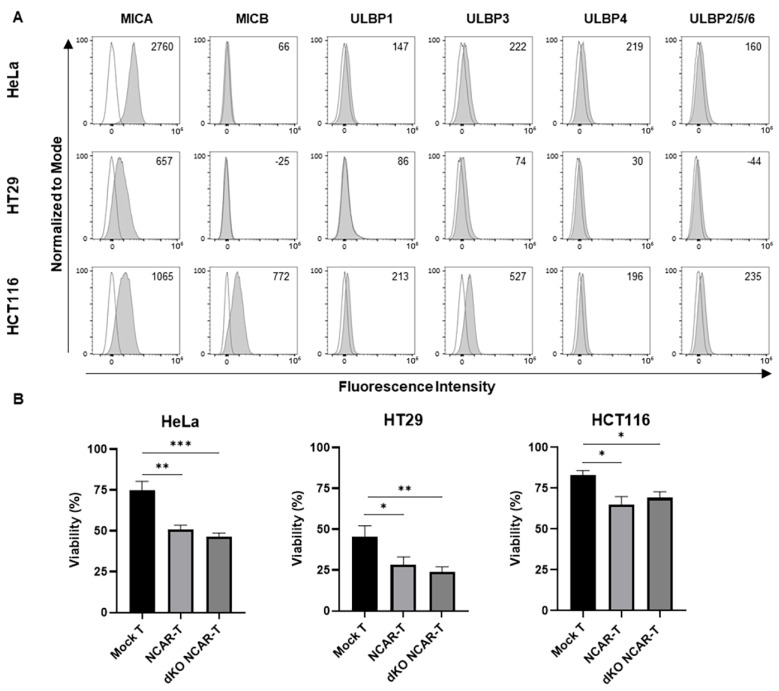
In vitro antitumor effect of the allogeneic NKG2D CAR-T cells against solid tumors: (**A**) Expression of the NKG2D ligands (MICA, MICB, ULBP1, ULBP3, ULBP4 and ULBP2/5/6) in cervicouterine (HeLa), and colorectal (HT29 and HCT116) tumor cell lines. (Black: Unstained cells; Grey: Stained cells). The numbers indicate the Median Fluorescence Intensity (MFI). (**B**) Viability percentage of HeLa, HCT116 and HT29 tumor cells after co-culture for 72 h with mock T cells (Mock T), NKG2D CAR-T cells (NCAR-T) and double-knockout NKG2D CAR-T cells (dKO NCAR-T) manufactured with IL-2 supplementation (effector-to-target ratio of 4:1). Data are shown as mean ± SEM (*n* = 5 independent experiments with different donors, technical quadruplicates). Statistical significance is represented as: * *p* < 0.05, ** *p* < 0.01, *** *p* < 0.001.

To study the efficacy of our allogeneic NKG2D CAR-T cell prototype, we tested its in vitro antitumor activity against potential cancer targets. First, we determined the expression of NKG2D-L in the tumor cells to be used in the cytotoxicity assay: human cervical cancer HeLa cells and colorectal cancer HCT116 and HT29 cells. We observed that at least one of the NKG2D-L was expressed in each tumor cell line, with the MICA ligand being the most commonly expressed in all the tumor cell lines assessed. (Figure 3A). MICB and ULBP3 were highly expressed in HCT116 cells, but were almost not expressed in the rest of the tumor cell lines. Regarding ULBP1, ULBP2/5/6 and ULBP4 ligands, they showed very low expression in all tumor cell lines.

To perform the cytotoxicity assay, mock T cells, NKG2D CAR-T cells and double-knockout NKG2D CAR-T cells (at day 11 after T cell activation) were cocultured with each tumor target cell line for 72 h at an E:T ratio of 4:1 to evaluate the in vitro antitumor effect by quantifying the decrease in GFP expression. The results showed that the double-knockout NKG2D CAR-T cells had a significantly higher antitumoral effect than the mock T cells when cocultured with all the cell lines assayed (HeLa, HCT116 and HT29) (Figure 3B). Moreover, the effects of double-knockout NKG2D CAR-T cells, despite being genetically modified to eliminate TCR expression, did not significantly differ from those of NKG2D CAR-T cells (TCR+). These results indicate the CAR-specific antitumor activity of the novel allogeneic CAR-T cell prototype.

### 3.4. Effect of Cytokine Supplementation on TRAC and B2M Gene Knockouts Using CRISPR/Cas9 Technology

**Figure 4:**
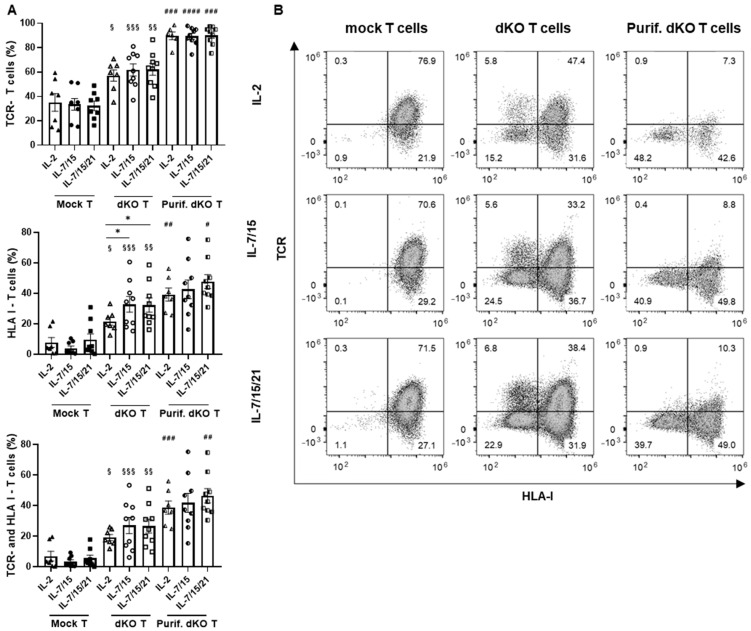
Comparison of the efficiency of TCR and HLA class I knockout by CRISPR/Cas9 in T cells cultured with different interleukin conditions: (**A**) Percentage of TCR-negative, HLA-I-negative or both TCR-negative and HLA-I-negative T cells in mock T cells (Mock T), double-knockout (*TRAC* and *B2M* genes) T cells before CD3-negative enrichment (double-knockout T cells, dKO T) and double-knockout T cells after CD3-negative enrichment (purified dKO T cells, Purif. dKO T) in culture supplemented with IL-2 (*n* = 7 independent experiments with different donors), IL-7/IL-15 (*n* = 9 independent experiments with different donors) or IL-7/IL-15/IL-21 (*n* = 9 independent experiments with different donors), 3 days after genetic modification (day 6; left). TCR−: TCR negative. HLA-I−: HLA-I negative. (**B**) Representative dot plots of TCR and HLA-I complex knockout analysis by flow cytometry (right). Data are shown as mean ± SEM. Statistical significance between mock T cells and dKO T cells is represented as: ^§^ *p* < 0.05, ^§§^ *p* < 0.01, ^§§§^ *p* < 0.001, and between dKO T cells and Purif. dKO T cells, as ^#^ *p* < 0.05, ^##^ *p* < 0.01, ^###^ *p* < 0.001, ^####^ *p* < 0.00001. Statistical significance between the different interleukin conditions is represented as * *p* < 0.05.

Given the relationship between a high proportion of early memory T cells and effective in vivo activity in CAR-T cell products, we aimed to increase the proportion of the Tscm cell subset in the double-knockout NKG2D CAR-T cells by comparing the conventional culture using IL-2 with the culture with different cytokine combinations associated with early memory T-cell enrichment, IL-7/-15 and IL-7/-15/-21. We analyzed the potential effect of these cytokine combinations in every step of the manufacturing process of the allogeneic NKG2D CAR-T cell prototype. Firstly, we determined the efficiency of the knockout of the *TRAC* and *B2M* genes after using the different cytokine supplementations. As illustrated in Figure 4, the knockout was efficient in all cases, and the results were similar for the different IL supplementations, except for HLA-I knockout with IL-2, whose efficiency was significantly decreased compared to the IL combinations.

However, after the TCR-negative enrichment process (by eliminating the CD3+ cells through magnetic bead selection), there were no differences in the proportion of genetically modified cells, including HLA-I-negative T cells. The proportion of TCR-negative T cells accounted for about 90% (89.7 ± 3.1% with IL-2, 89.3 ± 2.4% with IL-7/-15, and 90.16 ± 2.5% with IL-7/-15/-21), and the double-negative (TCR−/HLA-I−) T cells accounted for about 40% (38.7 ± 4.3% with IL-2, 41.8 ± 8.2% with IL-7/-15, and 46.4 ± 4.7% with IL-7/-15/-21) with all the IL supplementations.

In addition, to analyze potential genetic alterations due to the manufacturing process that could compromise product safety, G-banding karyotype analyses were performed in CRISPR/Cas9 gene-edited T cells and corresponding controls from four different healthy donors (Figure S2). No chromosomal aberrations were observed in metaphase spreads of edited T cells compared to wild-type T cells.

### 3.5. Comparison of the Characterization of Allogeneic NKG2D CAR-T Cells Produced with Different Cytokine Supplementations

**Figure 5:**
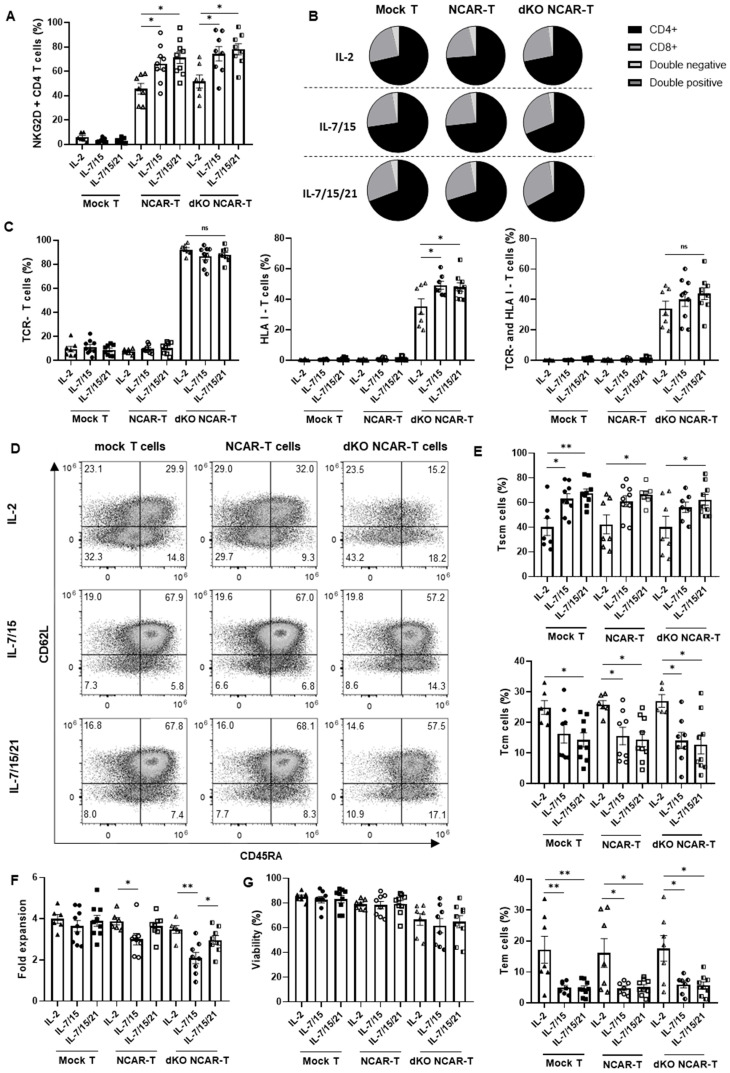
Effect of interleukin supplementation in the generation and characteristics of the allogeneic NKG2D CAR-T cells: (**A**) Percentage of NKG2D CAR expressing cells in mock T cells (Mock T), NKG2D CAR-T cells (NCAR-T) and double-knockout NKG2D CAR-T cells (dKO NCAR-T). (**B**) Distribution of T cell populations according to CD4 and CD8 expression in the mock T cells, NKG2D CAR-T cells and double-knockout NKG2D CAR-T cells. (**C**) Percentage of TCR-negative, HLA-I-negative or both TCR-negative and HLA-I-negative T cells in mock T cells, NKG2D CAR-T cells and double-knockout NKG2D CAR-T cells. (**D**) Representative dot plots of T-cell memory subpopulation analysis by flow cytometry. (**E**) Comparison of memory subpopulations of mock T cells, NKG2D CAR-T cells and double-knockout NKG2D CAR-T cells. (**F**) Fold expansion of mock T cells, NKG2D CAR-T cells and double-knockout NKG2D CAR-T cells for 4 days of culture from NKG2D-CAR transduction (from day 7 to 11). (**G**) Viability of mock T cells, NKG2D CAR-T cells and double-knockout NKG2D CAR-T cells. Measurements were performed on day 11 after manufacturing initiation, and the different assayed interleukin supplementations were IL-2 (*n* = 7 independent experiments with different donors), IL-7/-15 (*n* = 9 independent experiments with different donors), and IL-7/-15/-21 (*n* = 9 independent experiments with different donors). Tn: Naïve T cells (CD62L+ CD45RA+ CD95-). Tscm: Stem cell memory T cells (CD62L+ CD45RA+ CD95+). Tcm: Central memory T cells (CD62L+ CD45RA− CD95+). Tem: Effector memory T cells (CD62L− CD45RA− CD95+). Teff: Effector T cells. (CD62L− CD45RA+ CD95+). Data are shown as mean ± SEM. Statistical significance between the different interleukin conditions is represented as * *p* < 0.05, ** *p* < 0.01.

We compare the effect of the different cytokine conditions (IL-2, IL-7/-15 or IL-7/-15/-21) in the generation of the allogeneic NKG2D CAR-T cells and the product characteristics. We observed that the proportion of CAR-positive T cells after lentiviral transduction was significantly higher in the double-knockout NKG2D CAR-T cells manufactured in medium supplemented with IL-7/-15 (74.46 ± 5.9%) and IL-7/-15/-21 (78.1 ± 4.6%) compared to that obtained using IL-2 supplementation (53.16 ± 5.7%), according to analysis of CD4+ T cells (Figure 5A). A similar trend was observed when analyzing the NKG2D expression change in CD8+ T cells (Figure S3). Regarding the CD4/CD8 ratio, there were no significant changes in the CD4+ and/or CD8+ proportions among the CAR-T cells produced in the different conditions, ranging from 60% to 75% CD4+ T cells (Figure 5B).

We also determined, at the end of the manufacturing process (day 11), the percentages of TCR and HLA-I-negative cells to confirm that they were maintained throughout the process, and we observed that the results were similar to those from day 6. Moreover, the percentages of TCR-negative and double-negative T cells were similar across all the IL combinations (Figure 5C).

Since different IL supplementations are reported to affect the percentage of the memory subpopulations of T cells, we also measured the expression of T-cell memory markers by flow cytometry (Figure 5D). As can be seen in Figure 5E, the proportion of Tscm was significantly enriched in the double-knockout NKG2D CAR-T cells cultured with IL-7/-15/-21 (62.2 ± 4.3%) compared to those cultured with IL-2 supplementation (40.2 ± 7.8%). The same difference was also observed in NKG2D CAR-T cells and mock T cells. In contrast, the Tcm subset decreased significantly in the double-knockout NKG2D CAR-T cells cultured with IL-7/-15 and IL-7/-15/-21 in comparison with those cultured with IL-2. Concerning late memory T cells, the Tem percentage was also significantly higher in the product cultured with IL-2 compared to the one obtained with the other IL combinations.

Furthermore, we studied whether there was any difference in the proliferation capacity between the products manufactured with the different IL supplementations. Although significant reductions in both fold change and viability were observed in the double-knockout NKG2D CAR-T cells compared to NKG2D CAR-T cells and mock T cells in several IL conditions, IL-2 and IL-7/-15/-21 supplementations provided to the double-knockout NKG2D CAR-T cells a higher cell proliferation capacity in comparison with IL-7/-15 supplementation (Figure 5F), while the cell viability obtained with the different IL conditions was similar (Figure 5G).

To further study the results obtained on cell proliferation and viability, we also examined the potential cell fratricide by measuring IFN-γ release. As can be observed in Figure S4, both double-knockout NKG2D CAR-T cells and NKG2D CAR-T cells showed significantly higher IFN-γ release than mock T cells, with the double-knockout NKG2D CAR-T cells being the condition with the highest effect, suggesting an increased level of fratricide in these cells.

Moreover, to evaluate the risk of allorejection and GvHD of the double-knockout NKG2D CAR-T cells after administration, we conducted a two-way mixed lymphocyte reaction (MLR) in both allogeneic and autologous settings (see Supplementary Materials and Methods). The results showed that the IFN-γ levels in the coculture of double-knockout NKG2D CAR-T cells and allogeneic PBMCs were significantly lower than those in the coculture of NKG2D CAR-T cells and allogeneic PBMCs and similar to those in the coculture of NKG2D CAR-T cells and autologous PBMCs (Figure S5).

### 3.6. Effect of the Different Cytokine Supplementations on the In Vitro Antitumor Activity of the Allogeneic NKG2D CAR-T Cells Against Solid Cancers

**Figure 6:**
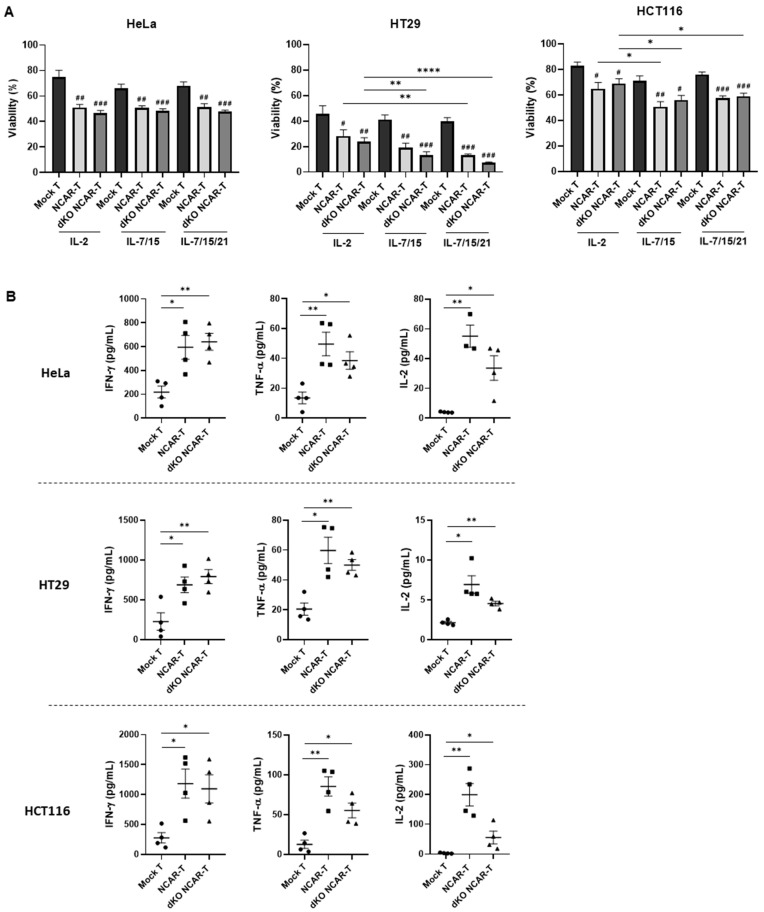
Differential in vitro antitumor effect against solid cancers of allogeneic NKG2D CAR-T cells manufactured with different interleukin supplementations: (**A**) Viability percentage of cervicouterine (HeLa) and colorectal (HCT116 or HT29) tumor cells after co-culture for 72h with mock T cells (Mock T), NKG2D CAR-T cells (NCAR-T) and double-knockout NKG2D CAR-T cells (dKO NCAR-T) cultured with IL-2, IL-7/IL-15 and IL-7/IL-15/IL-21 supplementations (effector-to-target ratio of 4:1). Data are shown as mean ± SEM (*n* = 5 independent experiments with different donors, technical quadruplicates). Statistical significance between mock T cells and NKG2D CAR-T cells or double-knockout NKG2D CAR-T cells was represented as ^#^ *p* < 0.05, ^##^ *p* < 0.01, and ^###^ *p* < 0.001. Differences between interleukin conditions are represented as * *p* < 0.05, ** *p* < 0.01, **** *p* < 0.0001. (**B**) Release of IFN-γ, TNF-α and IL-2 by mock T cells, NKG2D CAR-T cells and double-knockout NKG2D CAR-T cells, produced with IL-7/IL-15/IL-21 supplementation, after 72 h of co-culture with the different solid tumor cell lines (HeLa, HCT116 or HT29). Data are shown as mean ± SEM (*n* = 4 independent experiments with different donors, technical duplicates). Statistical significance is represented as * *p* < 0.05, ** *p* < 0.01.

To study whether the double-knockout NKG2D CAR-T cells produced with the different IL supplementations have different antitumor activity, we performed cytotoxicity assays against three different solid tumor cell lines (HeLa, HCT116 and HT29). The results showed that double-knockout NKG2D CAR-T cells, regardless of the IL supplementations, had a significantly greater antitumor effect than mock T cells when cocultured with HeLa, HCT116 and HT29 tumor cell lines (Figure 6A). Comparing the antitumor activity between the different ILs, we observed that the effect against cervicouterine HeLa cells was similar for all the conditions (approx. 50% cytotoxicity), while the effect against colorectal cancer cells (HT29 and HCT116) was higher with the double-knockout NKG2D CAR-T cell products manufactured with IL-7/-15 or IL-7/-15/-21 supplementations compared to those manufactured with IL-2. Moreover, HT29 cells showed higher sensitivity than HCT116 cells to this antitumor effect, obtaining >90% cytotoxicity in HT29 cells and approximately 40% cytotoxicity in HCT116 cells after treatment with double-knockout NKG2D CAR-T cells produced with IL-7/-15/21 supplementation. Interestingly, these results do not correlate with the differences in NKG2D ligand expression between these cell lines, since HCT116 expresses higher levels of the eight ligands. To further support the specific NKG2D-CAR activity, we compared the antitumor activity of the allogeneic NKG2D CAR-T cells against HeLa cells with or without the specific genetic modification to overexpress ULBP2 (detailed in Supplementary Materials and Methods) [33]. As can be seen in Figure S6, the cytotoxicity against the ULBP2-overexpressing HeLa cell line was significantly higher.

To confirm the antitumor activity of the double-knockout NKG2D CAR-T cells produced, we also studied the release of proinflammatory cytokines after coculturing them with the tumor target cells HeLa, HCT116 and HT29. To that end, we quantified the expression of IFN-γ, TNF-α and IL-2 by ELISA after 72 h of coculturing (time point setup illustrated in Figure S7). The results showed that NKG2D CAR-T cells and the double-knockout NKG2D CAR-T cells secreted significantly higher amounts of cytokines than the mock T cells in all cases studied (Figure 6B), thus confirming the specific antitumor activity of the NKG2D CAR-T cells against the solid tumor cells.

## 4. Discussion

Commercialized CAR-T therapy has achieved impressive results in patients with hematological malignancies. Indeed, axicabtagene ciloleucel and tisagenlecleucel therapies have obtained, according to real-world experience, an overall response rate of 80% and 66% and a complete response rate of 60% and 42%, respectively, in relapsed/refractory diffuse large B cell lymphoma [34]. However, currently commercialized CAR-T cell therapies have some limitations, mainly because they are designed for autologous use, their CARs are directed to one specific antigen and none of them are indicated for solid tumors.

The aim of this work was to develop a novel CAR-T-cell therapy for allogeneic use with efficacy against a wide variety of cancers, including solid tumors. To that end, we produced double-knockout (TCR−/HLA-I−) T cells and genetically modified them to express the NKG2D-CAR. In order to optimize the manufacturing process, three different conditions of interleukins (IL-2, IL-7/-15 and IL-7/-15/-21) were tested in the culture medium to determine their effects on both the genetic modification procedures and the final product characteristics.

Most of the disadvantages associated with autologous therapies are overcome by the “off-the-shelf” therapies, although they must be modified to avoid GvHD and allorejection [5,10]. In this work, we applied CRISPR/Cas9 technology to simultaneously disrupt both *TRAC* and *B2M* genes, which are essential components for the expression of TCR and HLA class I surface molecules, preventing GvHD and allorejection of the allogeneic CAR-T cell product, respectively [11,35,36]. Although specific comparisons should be studied in more depth, among the different strategies for allogeneic CAR-T-cell development, CRISPR/Cas9-based approaches are considered optimal due to their high flexibility that allows multiplex modifications, high efficiency, moderate labor and costs, and relatively easy standardization and scalability [10]. We obtained about 90% of TCR-negative T cells and more than 45% of double-knockout T cells by our manufacturing process with the IL-7/-15/-21 supplementation. This result is in the range of those obtained previously by other research groups, which range from 47% to 65% of the double-negative T-cell population [11,35,37]. Although CRISPR/Cas9 technology has been defined as a safe gene editing method, and it has even been used for engineered cells applied to human patients, including CAR-T cells [38], it is important to consider the risk of producing genetic alterations, especially with multiplex gene editing [39]. In this regard, karyotyping analyses have been reported as part of the quality control in the manufacturing process of CAR-T cells for clinical trials [40,41]. In this work, we used in silico prediction analyses during gRNA selection to identify and minimize potential off-target sites [42], and we selected the CRISPR technology based on nucleofection of Cas9-gRNA ribonucleoproteins to decrease the risk of off-target effects, which are more likely with strategies based on permanent expression of the genome editing complex. Moreover, previous works using these gRNAs did not observe off-target effects, or considered them very rare, when analyzing the top predictive off-target sites by experimental detection [35,43]. Additionally, we also analyzed the effect of the CRISPR/Cas9-based genetic modification by G-band karyotyping, and the results showed no chromosomal translocation, aneuploidy or other abnormalities that could compromise product safety. Nevertheless, additional in-depth analyses such as LAM-HTGTS, GUIDE-seq or low-pass WGS are warranted for genome-wide off-target detection, to further evaluate product safety [42].

On the other hand, to obtain a CAR-T-cell product suitable for clinical use, GMP-compliant reagents, processes, equipment and regulations must be taken into account (more information can be found in previous publications [44,45]). In our final GMP manufacturing process, an additional step should be implemented to obtain a higher purity of double-knockout CAR-T cells. This is essential, especially to reduce the risk of GvHD mediated by the TCR, and would also be convenient to decrease the immune elimination of therapeutic cells that express HLA-I. It can be performed by applying some of the recently developed cGMP-compliant Fluorescence-Activated Cell Sorting (FACS) technologies or multi-stage magnetic selection [45].

Regarding the NKG2D-CAR, the fact that it is able to recognize eight different ligands (MICA, MICB, y ULBP1–6) [19] provides multiple advantages, since it may be a solution for the heterogenicity that characterizes solid tumors [46], and it is less prone to antigen immune escape and, therefore, to therapy resistance and tumor relapses. Moreover, it has been reported that NKG2D CAR-T cells can impair the immunosuppressive tumor microenvironment by targeting the tumor-infiltrating myeloid-derived suppressor cells and immunosuppressive regulatory T cells, which overexpress the NKG2D target ligands [23,47]. Consequently, T-cell infiltration and activation is enhanced, and the probability of T-cell exhaustion is reduced, thus lowering the risk of treatment failure [18]. Furthermore, by disrupting the tumor microenvironment, they also have an anti-angiogenic effect [18], which is enhanced by the recognition of the endothelial cells of the tumor neovasculature by the NKG2D CAR-T cells [21]. In addition, previous studies have reported that NKG2D CAR-T therapy, in autologous use, is safe and effective in vitro and in mouse models [20,23], and there are ongoing clinical trials that study the effect of this kind of autologous therapy in acute myeloid leukemia, multiple myeloma, myelodysplastic syndrome colorectal cancer, ovarian cancer, bladder cancer, pancreatic cancer, glioblastoma, medulloblastoma, hepatocellular carcinoma and triple-negative breast cancer [24]. Furthermore, this CAR has recently been employed in mouse models for the treatment of virus-induced tumors, such as Epstein–Barr virus-associated lymphoproliferative disorder, in which, in addition to the elimination of tumor cells, it reduces viral dissemination through the removal of infected cells [48]. In the context of cancer, it is important that several tumor types show NKG2D ligand shedding as a mechanism of immunosuppression [49], so its inhibition should be considered when applying therapies based on the NKG2D-CAR. To date, there is only one clinical trial with allogeneic NKG2D CAR-T cells, whose CAR is co-expressed with a TCR inhibitor [50], although allogeneic therapies based on NKG2D CAR-NK cells have also been developed [51]. Moreover, in contrast to other NKG2D-CAR designs that incorporate the full-length NKG2D receptor [24], the CAR used in this study does not require endogenous DAP10 for stabilization. In our study, we obtained high expression of the NKG2D-CAR through lentiviral transduction and, interestingly, the transduction efficiency was significantly higher with IL-7/15 or IL-7/-15/-21 culture conditions (more than 70% in double-knockout CAR-T cells) than with IL-2 supplementation (around 50%). In general, the results of CAR expression were comparable to other studies of NKG2D CAR-T cells, which ranged from 45 to 87% CAR-positive cells [52,53,54], and this percentage would be suitable for clinical use as the minimum of the release criteria established for product infusion is usually around 10-15% CAR-positive T cells [55,56]. Regarding the effects of IL supplementation, Du et al. previously showed that the addition of IL-21 significantly increased transduction efficiency. However, they observed this increase when comparing IL-7/-15 and IL-7/-15/21 combinations, while our results show similar results with both combinations [29].

One aspect that is becoming relevant in the CAR-T-cell production process is the enrichment of early memory T cells. This is important because it has been observed that a CAR-T product with a higher proportion of Tscm cells has greater in vivo persistence, tumor infiltration and antitumor efficacy [57]. Furthermore, this cell subtype is also less likely to induce neurotoxicity and cytokine release syndrome, thus having a safer profile for the patient [58]. We have developed an allogeneic NKG2D CAR-T-cell prototype with a high percentage of Tscm (the most prevalent T-cell subset in the product) and demonstrated that the supplementation with IL-7/-15 or IL-7/-15/-21 was better than the IL-2 supplementation for Tscm enrichment (approximately 60% versus 40% of Tscm cells). Accordingly, previous studies reported a higher amount of Tscm cells when T cells were cultured with IL-7/-15 compared to culturing with IL-2 [59,60]. We also found that the results were not affected by the genetic modifications of the manufacturing process, since the same results were observed in unmodified (mock) T cells. Moreover, although we did not see any significant difference between IL-7/-15 and IL-7/-15/-21, some researchers found that IL-7/-15/-21 increased the CD45RA+ CCR7+ T cells [29] and Tscm under sort CD3/CD28 stimulation (48h) [61] compared to IL-7/-15 supplementation. In addition to the memory phenotype, the analysis of T-cell exhaustion markers such as PD-1, CTLA4, TIM-3, or LAG-3 would contribute to better estimate CAR-T-cell persistence and durability of therapeutic response after patient administration [62]. Focus on memory subpopulations, Ibáñez-Navarro et al. have studied the use of CD45RA− T cells (Tcm and Tem) with NKG2D-CAR as another approach for allogeneic treatment, due to their lower alloreactivity [52].

Another factor that is susceptible to variation in T-cell culture is the CD4/CD8 ratio. In this regard, it has been reported that both subpopulations are relevant for the efficacy of CAR-T therapy, since each one has different properties: CD4+ CAR-T cells induce host immune activation to a greater extent, while CD8+ cells have a higher direct antitumor effect [63,64]. In contrast to previous studies [65], our CAR-T manufacturing process did not induce any shift in the CD4/CD8 ratio during T-cell culture, which remained stable from the starting material to the final product. Additionally, we have not seen any difference in the CD4/CD8 ratio when using the different IL supplementations tested, which agrees with the results from other authors [29].

Regarding the proliferation, we observed that the allogeneic NKG2D CAR-T cells showed significantly less expansion with the IL-7/-15 supplementation in comparison with supplementing with IL-7/-15/-21. This might be explained by the unique property of IL-21 of primarily activating STAT3, rather than STAT5 [60]. Moreover, the IL-7/-15/-21 combination induced the same proliferation capacity as IL-2. Similar results were also seen in other studies [29,61]. On the other hand, it has been previously described that the activated T cells transiently express the NKG2D ligands, leading to self-killing during manufacturing [66,67]. According to IFN-γ culture levels, we have observed that NKG2D-CAR-expressing cells can undergo fratricide. However, interestingly, allogeneic NKG2D CAR-T cells showed significantly higher IFN-γ release than NKG2D CAR-T cells, which could be due to the induction of NKG2D ligand expression as a consequence of the knockout process. Regarding cell expansion, although no significant differences were detected, a trend toward reduced expansion was observed in the allogeneic NKG2D CAR-T cells. In the evaluation of cell viability, we observed a slight decrease in both NKG2D CAR-T cells and allogeneic NKG2D CAR-T cells compared to mock T cells. Although the differences are small, the decreased cell expansion and viability of NKG2D-CAR-expressing cells could indicate that fratricide is occurring to some extent, which could be mitigated by the addition of a phosphoinositol-3-kinase inhibitor or a NKG2D-blocking antibody during product manufacturing [67], or by including in the CAR-T-cell design a specific shRNA that simultaneously targets MICA and MICB, which are the key NKG2D ligands expressed in activated T cells [68].

The second-generation NKG2D-CAR used in the present work has previously been used to modify T cells to target human ovarian [32] and triple-negative breast [69] cancer cells, as well as to generate CAR-NK cells against multiple myeloma [22], obtaining in both cases significant antitumor activity in vitro and in vivo. In our study, we demonstrated that the allogeneic NKG2D CAR-T cells have a significant cytotoxic effect against human cervical (HeLa) and colorectal (HCT116 and HT29) cancer cells, despite the relatively low expression levels of NKG2D ligands, except for MICA. On the other hand, it has been reported that HT29 and HCT116 have a differential sensitivity to cytokines such as IFN-ɣ [70], which might indicate that their differences in sensitivity to the CAR-T cells are due in part to the cytokine release derived from CAR-T-cell fratricide. Moreover, it should be noted that the HT29 cell line also shows high sensitivity to mock T cells. Nevertheless, we demonstrated that the NKG2D CAR-T cells exert a specific cytotoxic effect against tumor cells, since the induced overexpression of a specific NKG2D ligand on the target cells significantly increases CAR-T cytotoxicity. Regarding the different IL supplementations, the antitumor effect was significantly higher when the CAR-T cells were produced with IL-7/-15 or IL-7/-15/-21 compared to those produced with IL-2, in the case of colorectal cancer. Therefore, among the three different culture conditions, IL-7/-15/-21 showed the best result considering also Tscm cell subset proportion, cell proliferation and in vitro antitumor activity.

Although the lack of in vivo results is a limitation of this work and animal experiments should be performed to evaluate the safety of the novel CAR-T prototype and to validate these in vitro results, we expect to observe similar differences between the different interleukin conditions, since CAR-T cells produced with IL-2 showed less proportion of early memory T cells than the other interleukin combinations, and previous studies showed better in vivo expansion and antitumor activity with an earlier memory T-cell population [59] and, when the mouse model was re-challenged, therapeutic T cells with a higher proportion of early memory T cells showed higher persistence with a lower exhaustion profile than those with a more differentiated phenotype [58]. Regarding the type of tumors used, human cervical and colorectal tumor samples have been previously characterized, revealing that the majority of the NKG2DL were expressed [71,72], and indicating the clinical applicability of our prototype.

Supporting the antitumor activity, significantly higher levels of cytokine secretion (TNF-α, IFN-γ, IL-2) were observed after coculturing CAR-T cells with tumor cells, in comparison with mock T cells. Regarding IL-2, the levels were higher when coculturing with HeLa and HCT116 cells than with HT29 cells. Since IL-2 has been reported to be associated with the onset of cytokine release syndrome (CRS) [73], this effect should be closely monitored during further product development. Furthermore, our results suggest that the double disruption of the *TRAC/B2M* genes did not negatively affect the cytotoxic activity of the product, since the percentage of tumor cell viability after coculturing was similar to that obtained with CAR-T cells that had not undergone the double knockout. Accordingly, we have also observed no significant difference in cytokine secretion between the double-knockout NKG2D CAR-T cells and the NKG2D CAR-T cells. Similarly, Ren et al. showed that TCR-/HLA-negative CAR-T cells have equivalent antitumor activity and cytokine secretion to CAR-T cells, and also have similar in vivo tumor regression, proliferation and CAR-T-cell engraftment without developing GvHD [11]. In addition, some allogeneic CAR-T therapies have been tested in clinical trials, demonstrating their efficiency and safety [74,75].

Although we did not observe major functional disadvantages in vitro derived from TCR knockout besides a slight decrease in cell viability, in accordance with other investigations [43], in vivo studies have reported a lower persistence of TCR-knockout CAR-T cells [76], which would make it necessary to consider repeated dosing regimens or the application of the CAR-T-cell product as a bridge-to-transplant therapy in specific tumors. Nevertheless, there are also studies that claim that the effectiveness of TCR-knockout cells in prolonging survival on mouse tumor models [11,43], final memory phenotype [43] and CAR-T-cell engraftment [11,43] are similar to TCR-positive CAR-T cells, even after tumor re-challenge [43].

Another important point is the contribution of both HLA-I and TCR knockouts to reducing the risk of allorejection and GvHD, respectively. Our results showed reduced IFN-γ levels when the double-knockout NKG2D CAR-T cells are cocultured with allogeneic PBMCs, compared to the coculture of NKG2D CAR-T cells and allogeneic PBMCs, which is consistent with previous studies [7,37]. Moreover, the IFN-γ levels are similar to those of the NKG2D CAR-T cells cocultured with autologous PBMCs. However, HLA-I-deficient T cells are susceptible to elimination by host NK cells through missing self-response, and additional experiments should be performed to address this issue. In this regard, further modifications could be used to optimize our CAR-T prototype in order to avoid elimination by NK cells. Among the different strategies that have been recently explored to address this issue, additional modifications such as the induced expression of CD47, which have demonstrated to prevent both macrophage phagocytosis and NK cell effector responses, or non-classical HLA molecules such as HLA-E and HLA-G, which inhibit NK cell reactivity, could be suitable to obtain a less alloreactive product [77,78,79,80]. Alternative options include performing specific deletion of HLA-A and HLA-B instead of the B2M knockout, a strategy that avoids T-cell alloreactivity without preventing HLA-C and HLA-E expressions, thus inhibiting the NK cell attack [77,81]. Moreover, besides product modification, patient management could involve the administration of immunosuppressive drugs prior to treatment, which is currently the main strategy used in clinical settings to prevent immune rejection of these types of medicinal products [77].

## 5. Conclusions

In conclusion, while *TRAC* and *B2M* gene disruption with CRISPR/Cas9 technology [37] and the use of NKG2D-CARs [32] have been independently reported, our research uniquely integrated both approaches for the development of a novel CAR-T-cell product for allogeneic use with broad tumor specificity, including solid tumors. In addition, we demonstrated that the manufacturing process of these allogeneic NKG2D CAR-T cells is optimized by the use of the IL-7/-15/-21 combination, thus allowing us to obtain a product enriched in Tscm cells, with improved proliferation capacity and potent in vitro antitumor activity against solid tumors (cervicouterine and colorectal cancers). Moreover, the novel therapy is potentially useful for all cancer types that express the NKG2D ligands, which account for more than 70% [22]. This work establishes the basis of a new “off-the-shelf” CAR-T-cell therapy that would be useful for the treatment of multiple tumor types in the clinical setting. Future research, including in vivo safety/efficacy testing and strategies to eliminate residual TCR-positive cells as essential next steps, is warranted to move forward with the development of this novel CAR-T-cell product.

## Figures and Tables

**Table 1:** gRNA target sequence primers.

Primer	Sequence (5′-3′)
TRAC_gRNA_Forward	5′-TAATACGACTCACTATAGTCAGGGTTCTGGATATCTG-3′
TRAC_gRNA_Reverse	5′-TTCTAGCTCTAAAACACAGATATCCAGAACCCTGA-3′
B2M_gRNA_Forward	5′-TAATACGACTCACTATAGGCGAGCACAGCTAAGGCC-3′
B2M_gRNA_Reverse	5′-TTCTAGCTCTAAAACTGGCCTTAGCTGTGCTCGC-3′

## Data Availability

The data of this study are available from the corresponding author upon request.

## Supplementary Materials

The following supporting information can be downloaded at https://www.mdpi.com/article/10.3390/cancers17193186/s1. Supplementary Materials and Methods. Figure S1: General gating strategy in flow cytometry analyses, Figure S2: Karyotype analysis, Figure S3: NKG2D expression in CD8-positive allogeneic NKG2D CAR-T cells manufactured with different interleukin supplementations, Figure S4: Cytokine release of allogeneic NKG2D CAR-T cells during culture, Figure S5: Cytokine release after coculturing allogeneic NKG2D CAR-T cells with autologous or allogeneic PBMCs, Figure S6: Cytotoxic effect of allogeneic NKG2D CAR-T cells against tumor cells genetically modified to overexpress a specific NKG2D ligand, Figure S7: Cytokine release kinetics of allogeneic NKG2D CAR-T cells in response to target cells.

## Abbreviations

The following abbreviations are used in this manuscript:

B2M	β-2 microglobulin
CAR	Chimeric antigen receptor
CRISPR	Clustered regularly interspaced short palindromic repeats
CRISPR/Cas9	CRISPR/Cas9 protein
CRS	Cytokine release syndrome
E:T	Effector-to-target
FACS	Fluorescence-Activated Cell Sorting
FBS	Fetal bovine serum
GvHD	Graft-versus-host disease
gRNAs	Guide RNAs
HLA	Human leukocyte antigen
HLA-I	HLA class I
IL	Interleukin
iPSC	Induced pluripotent stem cells
MFI	Median Fluorescence Intensity
MLR	Mixed lymphocyte reaction
NK	Natural killer
NKG2D	Natural-killer group 2 member D
NKG2D-L	NKG2D ligands
PBMC	Peripheral blood mononuclear cells
RNP	Ribonucleoprotein
TALEN	Transcription activator-like effector nucleases
Tcm	Central memory T cells
TCR	T-cell receptor
Teff	Effector T cells
Tem	Effector memory T cells
Tscm	Stem cell memory T cells
TU	Transducing units
ZFN	Zinc finger nucleases
