# Supplementary material for: A Novel Early Memory-Enriched Allogeneic NKG2D CAR-T Cell Therapy Based on CRISPR/Cas9 Technology for Solid Tumors
Source: Cancers (Basel). 2025 Sep 30;17(19):3186. doi: 10.3390/cancers17193186 (PMC12523248; doi:10.3390/cancers17193186)
Supplement: Supplementary file 1 [file cancers-17-03186-s001.zip › Aparicio et al. Supplementary Materials and Methods_DEF.pdf]

1. Supplementary Materials and Methods

1.1. Overexpression of NKG2D ligands by CRISPR-dCas9-based gene activation (CRISPRa) in HeLa cells

HeLa cells were genetically modified to specifically overexpress the NKG2D ligand ULBP2 using CRISPRa technology according to the methodology previously reported by Gonzalez-Vallinas *et al*. [1]. Firstly, the cells were lentivirally transduced with the pLenti-EFS-dCas9-VPR-PGK-Puromycin vector and subsequently selected with puromycin. These cells underwent a second lentiviral transduction with the pLKO-U6sgRNA-EF1α-GFP-P2A-Blasticidin vector, previously modified by the insertion of the fragment containing the sgRNA ULBP2-targeting sequence (Fwd: 5′-CACCGAGAGGAATATTCAGCGGCGG-3′; Rev: 5′-AAACCCGCCGCTGAATATTCCTCTC-3′), and genetically modified cells were selected with blasticidin.

To confirm gene overexpression, we performed RT-qPCR as previously described [1], using the specific primers for ULBP2 (Fwd: 5′-CTGGAGAATTACACACCCAAG-3′; Rev: 5′-CCCATCGAAACTGAACTGC-3′). Additionally, cell surface ligand overexpression was determined by flow cytometry.

1.2. Fratricide Assay

The potential CAR-T cell fratricide was assessed by ELISA determination of IFN-γ release by CAR-T cells (2×10^5) cultured in 96-well flat bottom tissue-culture plates during 72 h in RPMI 1640 Medium + GlutaMAX™-l supplemented with 10% FBS.

1.3. Two-way Mixed Lymphocyte Reaction (MLR) Assay

To evaluate alloreaction, we cocultured the CAR-T cells with autologous or allogeneic PBMCs, previously stained with the CellTrace™ Violet dye (C34557, ThermoFisher Scientific). The cells were cultured in 96-well flat bottom tissue-culture plates with RPMI 1640 Medium + GlutaMAX™-l supplemented with 10% FBS at a 1:1 ratio (8×10^4 cells). After 72 h, IFN-γ levels were measured by ELISA.

References

1. González-Vallinas, M.; Rodríguez-Paredes, M.; Albrecht, M.; Sticht, C.; Stichel, D.; Gutekunst, J.; Pitea, A.; Sass, S.; Sánchez-Rivera, F.J.; Lorenzo-Bermejo, J.; et al. Epigenetically Regulated Chromosome 14q32 MiRNA Cluster Induces Metastasis and Predicts Poor Prognosis in Lung Adenocarcinoma Patients. *Mol Cancer Res* **2018**, *16*, 390–402.
