# Supplementary material for: A Novel Early Memory-Enriched Allogeneic NKG2D CAR-T Cell Therapy Based on CRISPR/Cas9 Technology for Solid Tumors
Source: Cancers (Basel). 2025 Sep 30;17(19):3186. doi: 10.3390/cancers17193186 (PMC12523248; doi:10.3390/cancers17193186)
Supplement: Supplementary file 1 [file cancers-17-03186-s001.zip › Aparicio et al. Supplementary Figures S1-S7_DEF.pdf]

Supplementary Figures S1-S7

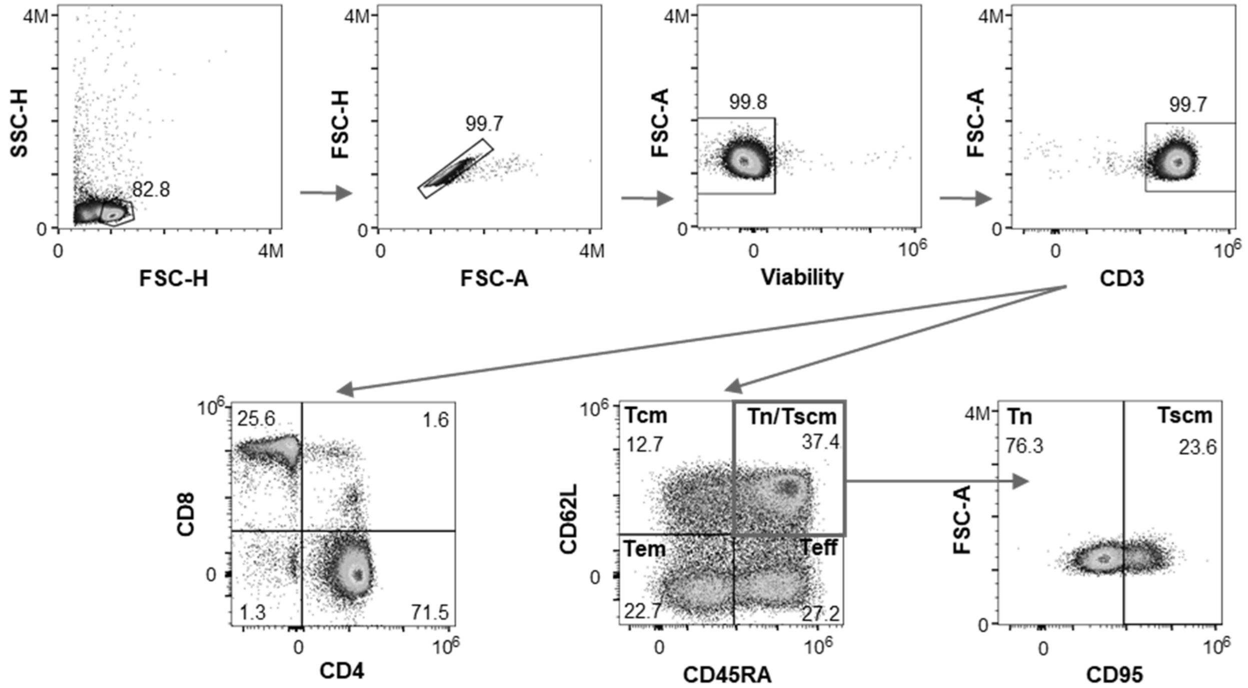

Figure S1. General gating strategy in flow cytometry analyses. Representative example of the general analysis of T cells (CD3+), memory subpopulations and CD4/CD8 ratio by flow cytometry. Firstly, debris and large particles are excluded with forward scatter (FSC) and side scatter (SSC); next, the doublets are excluded with FSC-Area (FSC-A) and FSC-Height (FSC-H); subsequently, the non-viable cells are excluded with the viability stain (positive population). The T cell population is selected according to CD3 expression before further quantifications. The CD4/CD8 ratio is determined by confronting the two markers (CD4 and CD8) in a dot plot and determining the different subpopulations: CD4-positive T cells (CD4+ CD8—), CD8-positive T cells (CD4— CD8+), double-positive T cells (CD4+ CD8+) and double-negative T cells (CD4— CD8—). To quantify T cell memory subpopulations, CD62L, CD45RA and CD95 markers are analyzed; a dot plot of CD62L and CD45RA defines the effector T cells (Teff; CD62L— CD45RA+), effector memory T cells (Tem; CD62L— CD45RA—), central memory T cells (Tcm; CD62L+ CD45RA—) and naïve T cells/ stem cell memory T cells (CD62L+ CD45RA+); to distinguish between the latter two populations, we use the CD95 marker: naïve T cells (Tn; CD62L+ CD45RA+ CD95—) and stem cell memory T cells (Tscm; CD62L+ CD45RA+ CD95+).

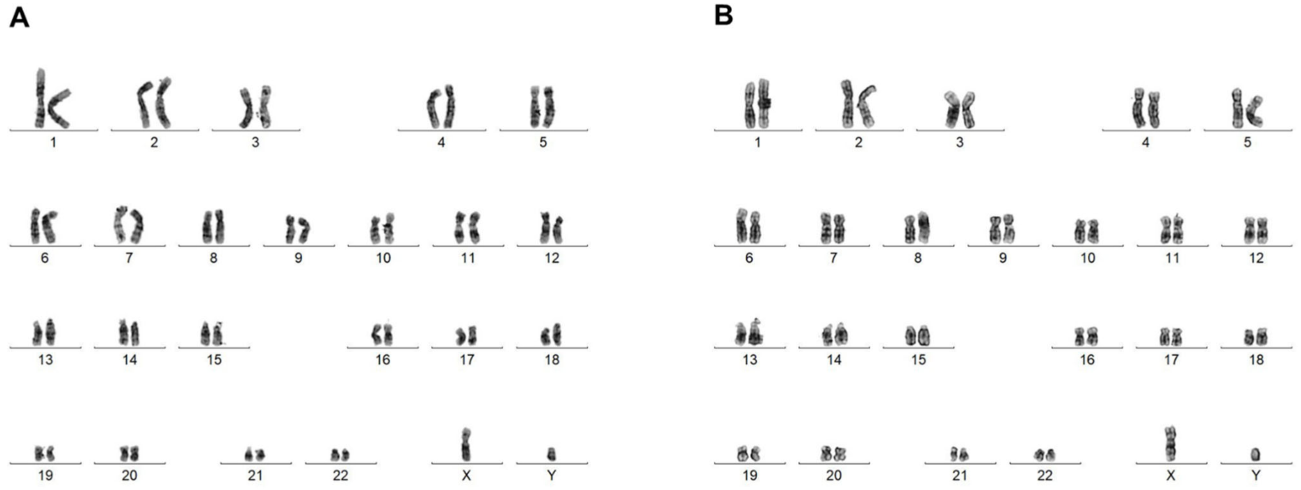

Figure S2. Karyotype analysis. Representative images of the karyotype analyses performed on wild-type T cells (**A**) and CRISPR/Cas9 gene-edited T cells (**B**) cultured with IL-7/IL-15/IL-21 supplementation (*n*=4 independent experiments with different donors). No chromosomal aberrations were observed.

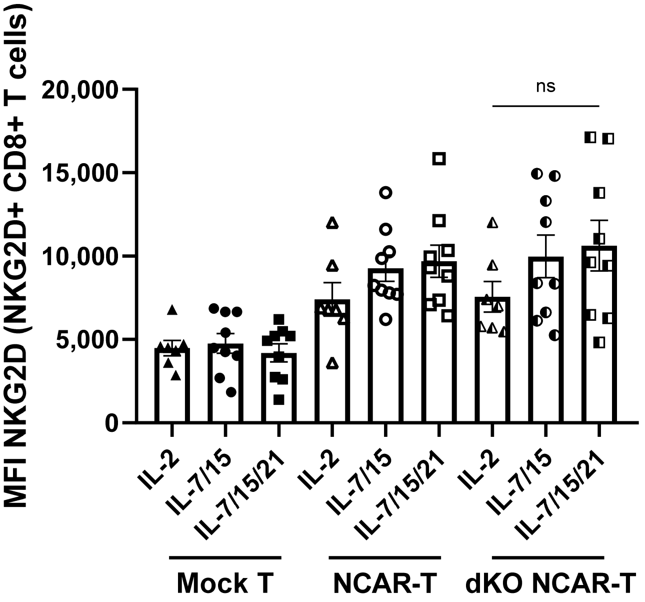

Figure S3. NKG2D expression in CD8-positive allogeneic NKG2D CAR-T cells manufactured with different interleukin supplementations. Median Fluorescence Intensity (MFI) of NKG2D in NKG2D- and CD8-positive mock T cells (Mock T), NKG2D CAR-T cells (NCAR-T) and double-knockout (*TRAC* and *B2M* genes) NKG2D CAR-T cells (dKO NCAR-T) at day 11 after the initiation of manufacturing with the interleukin supplementations IL-2 (n=7 independent experiments with different donors), IL-7/IL-15 (n=9 independent experiments with different donors), and IL-7/IL-15/IL-21 (n=9 independent experiments with different donors). Data are shown as mean ± SEM. Statistically significant differences were determined between the different interleukin conditions. ns: not significant.

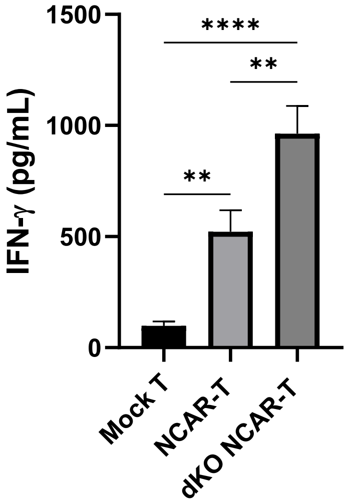

Figure S4. Cytokine release of allogeneic NKG2D CAR-T cells during culture. Release of IFN- γ by Mock T cells (Mock T), NKG2D CAR-T cells (NCAR-T) and double-knockout NKG2D CAR-T cells (dKO NCAR-T), produced with IL-7/IL-15/IL-21 supplementation, after 72 hours of culture. Data are shown as mean ± SEM (*n*=3, technical duplicates). Statistical significance between the different conditions was represented as: **p<0.01, ****p<0.0001.

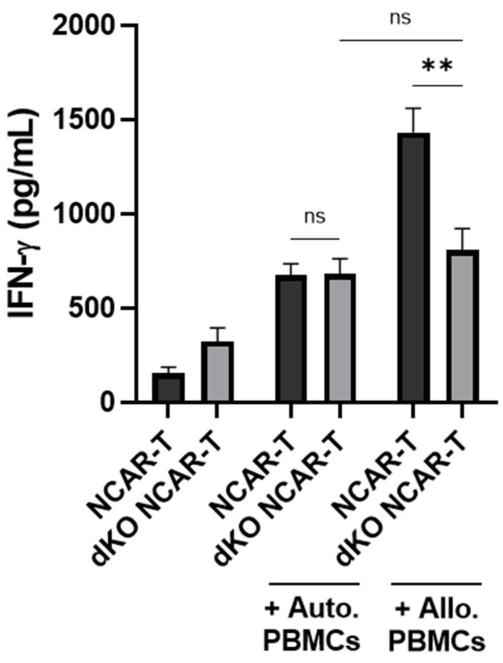

Figure S5. Cytokine release after coculturing allogeneic NKG2D CAR-T cells with autologous or allogeneic PBMCs. Release of IFN- γ after coculturing NKG2D CAR-T cells (NCAR-T) and double-knockout NKG2D CAR-T cells (dKO NCAR-T), produced with IL-7/IL-15/IL-21 supplementation, with allogeneic peripheral blood mononuclear cells (Allo. PBMCs) or autologous PBMCs (Auto. PBMCs) during 72 h. Data are shown as mean ± SEM (*n*=3, technical duplicates). Statistically significant differences between coculturing conditions were represented as: **p<0.01. ns: not significant.

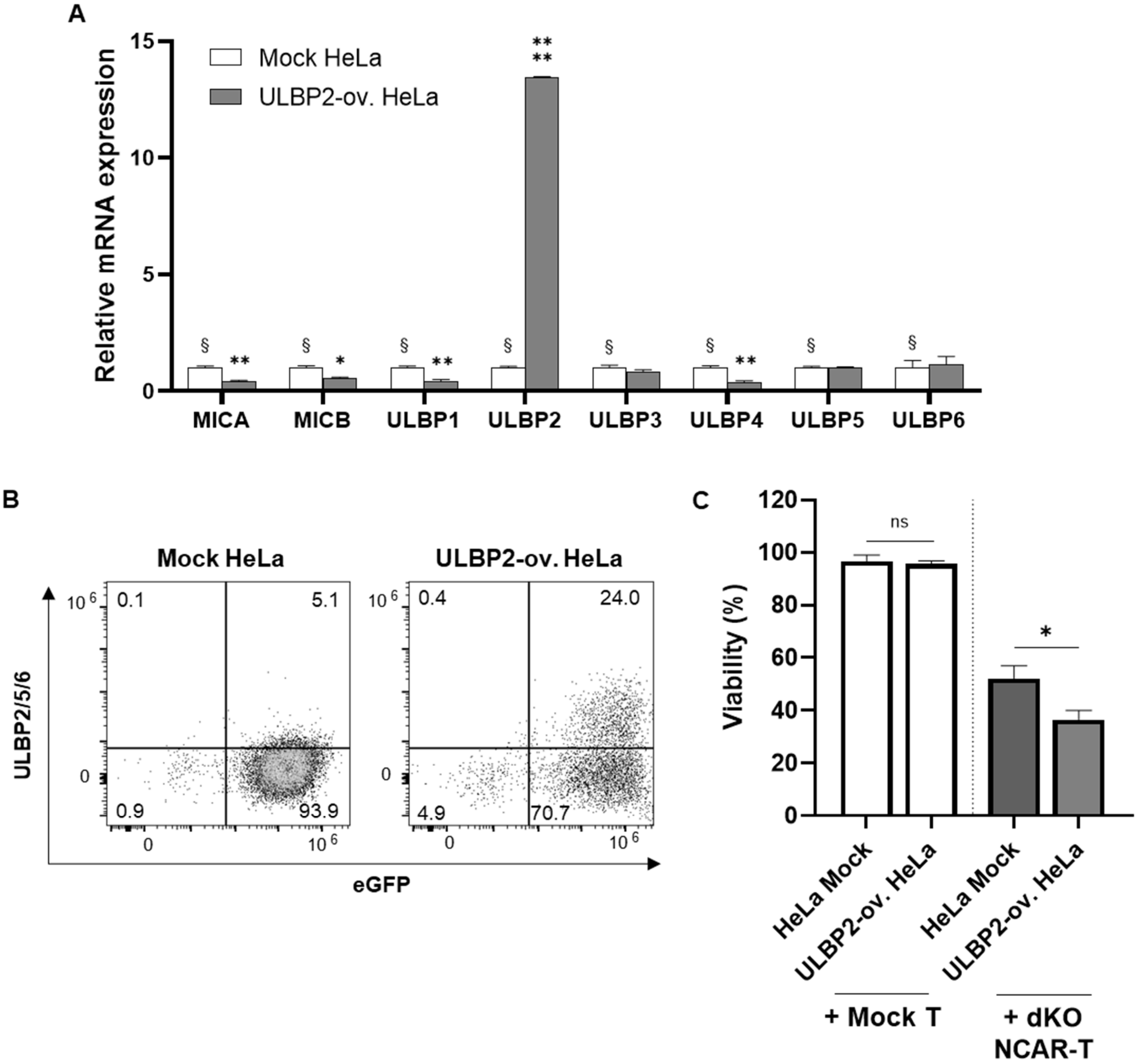

Figure S6. Cytotoxic effect of allogeneic NKG2D CAR-T cells against tumor cells genetically modified to overexpress a specific NKG2D ligand. (**A**) Relative gene expression of the eight NKG2D ligands in HeLa cells modified by CRISPR activation technology to overexpress ULBP2 (ULBP2-ov. HeLa) compared to mock HeLa cells (same genetic modification without sgRNA targeting sequence; Mock HeLa), which demonstrates specific overexpression of ULBP2 among the different NKG2D ligands. Data are shown as mean ± SEM (*n*=3 independent experiments). Statistically significant differences between Mock HeLa (control) and ULBP2-ov. HeLa are represented as: *p<0.05, **p<0.01, ****p<0.0001. § control. (**B**) Determination by flow cytometry of ULBP2/5/6 membrane expression and eGFP expression (genetic modification marker) in the mock HeLa cells and the ULBP2-overexpressing HeLa cells. (**C**) Cell viability of control (Mock HeLa) and ULBP2-overexpressing (ULBP2-ov. HeLa) cervicouterine tumor cells after coculturing for 72h with mock T cells (Mock T) and double-knockout NKG2D CAR-T cells (dKO NCAR-T) manufactured with IL-7/IL-15/IL-21 supplementation. Data are shown as mean ± SEM (n=2, technical quadruplicates). Statistically significant differences between the effects against control tumor cells and ULBP2-overexpressing tumor cells was represented as: *p<0.05. ns: not significant.

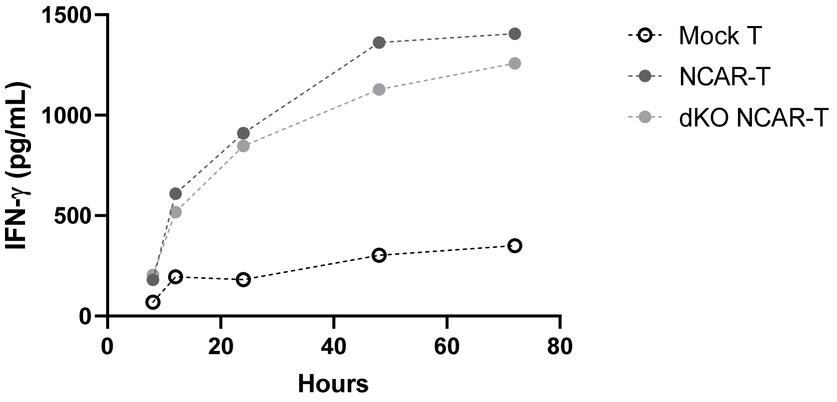

Figure S7. Cytokine release kinetics of allogeneic NKG2D CAR-T cells in response to target cells. Release of IFN-γ by Mock T cells (Mock T), NKG2D CAR-T cells (NCAR-T) and double-knockout (*TRAC* and *B2M* genes) NKG2D CAR-T cells (dKO NCAR-T), produced with IL-7/IL-15/IL-21 supplementation, after 8, 12, 24, 48 and 72 hours of co-culturing with the HCT116 colorectal cancer cell line (*n*=1, technical duplicates).
